# New Eco-Friendly Synthesized Thermosets from Isoeugenol-Based Epoxy Resins

**DOI:** 10.3390/polym12010229

**Published:** 2020-01-17

**Authors:** Quentin Ruiz, Sylvie Pourchet, Vincent Placet, Laurent Plasseraud, Gilles Boni

**Affiliations:** 1 ICMUB Institute, Université de Bourgogne Franche-Comté, UMR 6302 CNRS-UB, F-21000 Dijon, France; quentin_ruiz@etu.u-bourgogne.fr (Q.R.);; 2 FEMTO-ST Institute, Université de Bourgogne Franche-Comté, UMR 6174 CNRS-UFC-ENSMM-UTBM, Department of Applied Mechanics, F-25000 Besancon, France; vincent.placet@univ-fcomte.fr

**Keywords:** bio-based resins, epoxy thermosets, isoeugenol, crosslinking density, DMA

## Abstract

Epoxy resin plays a key role in composite matrices and DGEBA is the major precursor used. With the aim of favouring the use of bio resources, epoxy resins can be prepared from lignin. In particular, diglycidyl ether of isoeugenol derivatives are good candidates for the replacement of DGEBA. This article presents an effective and eco-friendly way to prepare epoxy resin derived from isoeugenol (BioIgenox), making its upscale possible. BioIgenox has been totally characterized by NMR, FTIR, MS and elemental analyses. Curing of BioIgenox and camphoric anhydride with varying epoxide function/anhydride molar ratios has allowed determining an optimum ratio near 1/0.9 based on DMA and DSC analyses and swelling behaviours. This thermoset exhibits a Tg measured by DMA of 165 °C, a tensile storage modulus at 40 °C of 2.2 GPa and mean 3-point bending stiffness, strength and strain at failure of 3.2 GPa, 120 MPa and 6.6%, respectively. Transposed to BioIgenox/hexahydrophtalic anhydride, this optimized formulation gives a thermoset with a Tg determined by DMA of 140 °C and a storage modulus at 40 °C of 2.6 GPa. The thermal and mechanical properties of these two thermosets are consistent with their use as matrices for structural or semi-structural composites.

## 1. Introduction

Epoxy thermosets result from a polymerization reaction between an epoxy resin and a hardener, mainly polyamine or acid anhydride. Although epoxy resins are more expansive than the unsaturated polyester resins, they play a key role in the market of paints, glue, varnishes or in the sectors of transport and building due to their high adhesive and mechanical properties [1].

Nowadays, diglycidyl ether of bisphenol A (DGEBA) represents 90% of the commercial epoxy resins. Its synthesis results from the reaction between two molecules of epichlorohydrin and a molecule of bisphenol A (BPA). However, the use of DGEBA has two major drawbacks: BPA used in its synthesis is petro-sourced and, secondly, BPA, an endocrine disruptor [2,3], is released during ageing of DGEBA based epoxy thermosets.

With the aim of avoiding BPA reactant and of favouring the use of bio resources, many studies have been carried out. For instance, because of their abundance and low toxicity, plant oils are one of the bio-based feedstocks frequently used. Recently oxidation of unsaturated triglycerides derived from soybean [4] or linseed [5] have led to epoxy precursors. Nevertheless, their long hydrocarbon chains do not provide for the resulting mechanical and thermal properties required for high-grade materials. Thus, in order to develop bio-based epoxy matrices for semi-structural and structural applications, epoxy precursors need to contain rigid cyclic groups or aromatic rings. In this context, lignin, a natural crosslinked phenolic polymer, is a good candidate as it is an abundant and accessible feedstock [6].

Epoxy resins derived from lignin are prepared according to two ways. Raw lignin can be epoxidized [7,8,9] and directly used as a bio-sourced epoxy resin. This way induces a variability of the resulting materials properties since the content of lignin in different types of biomass resources varies strongly. An alternative approach consists of lignin fragmentation [10,11,12] leading to a wide range of building blocks, such as vanillin [13,14,15], rosin [16], eugenol [17,18,19,20] and isoeugenol [21,22], which are then epoxidized.

Within this context, our research team has recently described both the synthesis of an epoxy monomer derived from isoeugenol, named as GlycidylEther Epoxy isoeugenol (GEEpiE) [21], and its curing with camphoric anhydride [22]. Behaviours of the resulting thermoset are consistent with its use as a bio-based epoxy matrix for structural composites. Such applications generally require, at the scale of the composite material, high strength and stiffness to weight ratios, damping properties, high durability, resistance to temperature impact and fire. For the matrix, it comes down to specific thermomechanical properties as well as specific rheological behaviour and a good affinity with the used fibers. Previous works [23] have demonstrated the ability of such thermoset systems to fulfil the requirements for structural composite applications, with high elastic properties and bending strength, high Tg and good flame-retardant properties (when using phosphate-based derivatives from isoeugenol). Nevertheless, GEEpiE synthesis needs a purification by chromatography on a silica column, which is time consuming and not environment-friendly. This last step is the major barrier to the development of the resin at a larger scale.

Thus, this work proposes a preparation improvement of the bio-based epoxy resin in order to make its scale-up possible. In particular, an extensive characterization of the reaction by-products by ^1^H NMR, ^13^C {^1^H} NMR, IR, mass spectrometry and elemental analysis, has allowed to suppress the chromatography purification step. Indeed, it has been shown that both the by-products obtained contain allylic groups easily converted into epoxide functions (ep) and leading thus also to diepoxy monomers. The resulting monomers mixture named as BioIgenox (BI) has subsequently been cured with bio-based camphoric anhydride and hexahydrophtalic anhydride. The resulting materials have been characterized by dynamic mechanical analysis (DMA) and differential scanning calorimetry (DSC) analyses and their swelling behaviour were measured. In order to optimize their thermal and mechanical properties, different formulations with epoxide function/anhydride molar ratio varying from 1/0.5 to 1/1, have been investigated. These results have then been discussed with the aim of developing a bio-based epoxy matrice for semi-structural or structural composites. Bending properties are also determined for the most promising ratio.

## 2. Materials and Methods

### 2.1. Materials

**Scheme 1:**
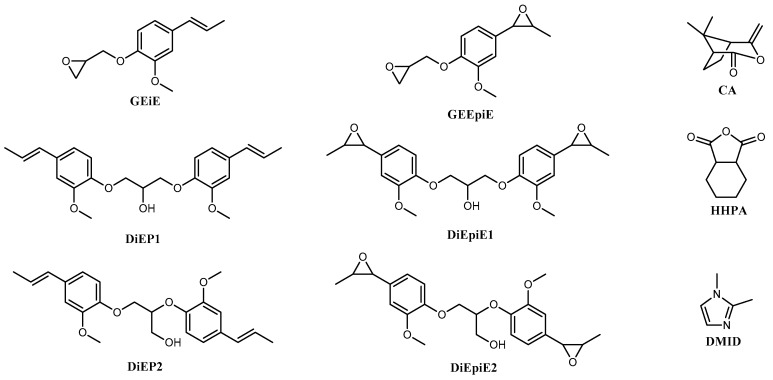
Molecular representations of reagents used in the resin manufacturing process. Left: Epoxy monomer precursors: BioIgenol (Glycidylether isoeugenol (GEiE), di isoeugenol propanol (DiEP1/DiEP2)). Center BioIgenox (Glycidylether epoxy isoeugenol (GEEpiE), di epoxy isoeugenol propanol (DiEpiE1/DiEpiE2)). Right: Anhydride hardeners: camphoric anhydride (CA), hexahydrophtalic anhydride (HHPA), catalyst: 1,2-dimethylimidazole (DMID).

Dichloromethane (DCM, analytical grade, Carlo Erba, Barcelona, Spain), acetone (analytical grade, Carlo Erba, Italy), sodium hydrogen carbonate (NaHCO_3_, ≥95% purity, Sigma-Aldrich, St. Louis, MO, USA), tetrabutylammonium hydrogen sulfate (Bu_4_NHSO_4_, ≥97% purity, Fluka, Chemie, GmbH, Buchs, Switzerland), epichlorohydrine (99% purity, Sigma-Aldrich), and Oxone^®^ (Sigma-Aldrich) were purchased and used as received. Natural isoeugenol (Sigma-Aldrich, 99%), diglycidyl ether of bisphenol A (DGEBA, Sigma-Aldrich, Steinheim, Germany), camphoric anhydride (CA, 98% purity, Alfa Aesar, Ward Hill, MA, USA), hexahydrophtalic anhydride (HHPA, 95% purity, Sigma-Aldrich) and 1,2-dimethylimidazole (DMID, 95% purity, Sigma-Aldrich) were purchased and used without any further purification. These reagents are depicted in Scheme 1.

### 2.2. Methods

#### 2.2.1. Characterizations and Instruments

FTIR spectra were recorded using a Bruker (Karlsruhe, Germany) Alpha spectrophotometer fitted with an ATR module ALPHA–P equipped with a mono-reflection diamond crystal in the 4000–375 cm^−1^ wavenumber range (32 scans at a spectral resolution of 4 cm^−1^).

^1^H and ^13^C {^1^H} nuclear magnetic resonance (NMR) spectra were obtained using a Bruker Avance III 500 MHz spectrometer (Karlsruhe, Germany) working at 500 and 125 MHz, respectively, at room temperature and using CDCl_3_ as solvent. External references were trimethylsilane (TMS) for ^1^H and ^13^C {^1^H} NMR.

Mass Spectrometry (MS) analysis was carried out using an HPLC Ultimate 3000 RSLC instrument from DIONEX (Sunnyvale, CA, USA) coupled through an electrospray ionisation (ESI) interface to an ion-trap AmaZon SL mass spectrometer from Bruker for MS detection. Methanol was used as the solvent.

Elemental analyses were performed on a CHNS/O Thermo Electron Flash EA 1112 Series apparatus.

Differential scanning calorimetry (DSC) analyses were performed on a TA Instruments (New Castle, DL, USA) Discovery DSC under nitrogen flow (50 mL min^−1^) with a sample mass of 10 ± 3 mg. To study the curing reaction, samples were heated from 20 °C to 200 °C at a heating rate of 5 °C min^−1^. The determination of glass transition temperatures (T_g_) is carried out on the samples cured in the oven. After a first heating from 20 °C to 180 °C, the T_g_ is determined on the second heating from 20 °C to 190 °C at a heating rate of 10 °C min^−1^ and using the TA Universal Analysis 2000 software.

Samples for dynamic mechanical analysis (DMA) tests were cut to the dimensions of 60 × 10 × 1.7 mm. Storage modulus (*E*′), loss modulus (*E*″) and loss factor (tan(*δ*)) were measured using a METRAVIB DMA50 apparatus at 5 °C intervals, under isothermal conditions. A stabilization of 3 min was maintained before each measurement to ensure a homogeneous temperature inside the specimens. The temperature was varied between 40 °C and 210 °C with a heating rate of 10 °C min^−1^ between each plateau temperature. The frequency of the excitation varied from 0.5 Hz to 5 Hz. A sinusoidal tensile displacement was applied on the sample with a peak-to-peak amplitude of 8 μm and a mean amplitude of 40 μm. For each batch, two specimens were systematically tested. A very good reproducibility of the measurements was observed. For reason of readability, in the present paper, results are presented in the Figure 4 for one specimen only.

The rubber elasticity theory defines the crosslinking density as [24]:

$$\rho =\frac{E^{\prime }}{\phi RT}$$

where *E*′ is the storage modulus at Tg + 30 °C (MPa), Φ is the front factor (approximated to 1 in the Flory theory), R is the gas constant (8314 cm^3^ MPa mol^−1^ K^−1^ and T is the absolute temperature at Tg + 30 °C (K). ρ is expressed in mol cm^−3^.

The swelling behaviour of the cured sample was studied by a solvent immersion method. The dry sample (weight w_0_) was immersed in THF at room temperature for 48 h. The weight of swollen sample (w_1_) was measured after wiping the surface with a filter paper. The swelling ratio (S) was calculated as follows:

S = 100 × (w_1_ − w_0_)/w_0_

Some of the cut specimens were also characterized under three-point bending tests following the ASTM D790 standard. Tests were performed using a universal testing machine MTS criterion 45 equipped with a 1 kN full-range load sensor. Three samples were tested at a constant crosshead displacement rate of 10 µm/s. The deflection was measured at mid-span, on the bottom face of the specimen, using a micrometre laser sensor. The support span was 40 mm. The ultimate stress ($\sigma_{max}$) and the strain at failure (ε) were determined from the load-deflection curves using the following equations:

$$\begin{array}{c} \sigma_{max}=\frac{3F_{max}L}{2bh^{2}}\\ \varepsilon =\frac{6hf}{L²} \end{array}$$

where $F_{max}$ is the maximum force applied to the sample, *L* is the distance between supports (span), *b* is the width of the sample, *h* is the thickness of the sample and f is the deflection.

The three-point bending modulus (*E*) was determined using the slope of the stress-strain curve in the strain range from 0.1% to 0.3%. The mean values and standard deviation were then calculated.

##### First Step: Synthesis of BioIgenox

**Scheme 2:**
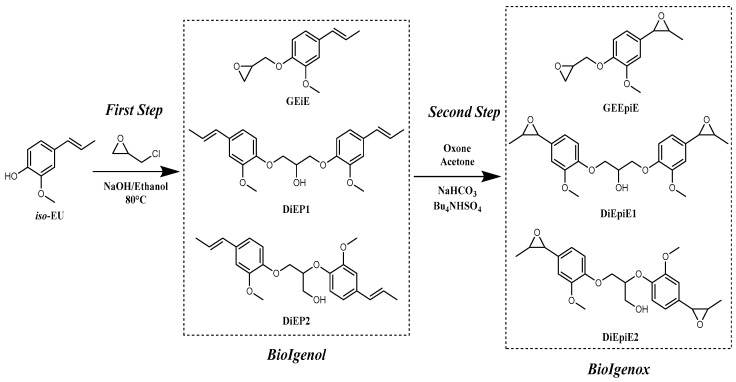
Synthetic pathway leading to BioIgenox from isoeugenol.

**Scheme 3:**
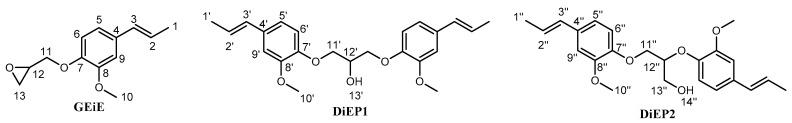
Molecular representation of BioIgenol.

BioIgenox (see Scheme 2) was synthesized according to published procedure [22] and modified as follows:

Isoeugenol (100 g, 0.609 mol) and a solution of NaOH (24.36 g, 0.609 mol) in ethanol (200 mL) are introduced in a round-bottom flask of 1000 mL equipped with a refrigerant and a dropping funnel and heated at 80 °C under argon atmosphere. Epichlorohydrin (220 mL, 2.38 mol) is added dropwise for 1 h and the mixture is stirred for 3 h. After cooling at room temperature, the mixture is filtered. The solid is rinsed with toluene (3 × 20 mL). The resulting organic solution is washed with water, saturated NaCl solution, and dried over anhydrous MgSO_4_ leading, after evaporation under vacuum, to produce an orange oil which solidifies to afford a cream-coloured solid, named BioIgenol (see Scheme 3), with a yield of 77%.

FTIR (ATR, cm^−1^): 3486 (O−H), 3019 (aromatic C−H), 1653 (C = C), 1601, 1583, 1509, 1464 (aromatic C = C), 910 (epoxy ring GEiE).

^1^H-NMR (500 MHz, CDCl_3_): *δ* ppm 1.88 (m, H1, H1′ et H1″), 2.74 and 2.89 (m, H13), 3.08 (m, H14″), 3.29 (m, H13′), 3.39 (m, H12), 3.57 (m, H12″), 3.88 (m, H10, H10′ et H10″), 4.02–4.28 (m, H11, H11′, H11″, H13″), 4.39 (m, H12′), 5,75 (m, H2, H2′, H2″ *trans*), 6.13 (m, H2, H2′, H2″ *cis*), 6.34 (m, H3, H3′, H3″), 6.82–6.95 (m, H5, H6, H9, H5′, H6′, H9′, H5″, H6″, H9″) (See Figure S1).

^13^C {^1^H} NMR (126 MHz, CDCl3): *δ* 150.0 (C8, C8′, C8″), 147.5 (C7, C7′, C7″), 132.6 (C4, C4′, C4″), 130.9 (C3, C3′, C3″), 124.6 (C2, C2′, C2″), 119.0 (C5, C5′, C5″), 114.6 (C6, C6′, C6″), 109.5 (C9, C9′, C9″), 71.8 (C12″), 71.5 (C11′), 70.7 (C12′), 70.3 (C11), 70.7 (C11″) 69.0 (C13″), 56.2 (C10, C10′, C10″), 50.6 (C12), 45.4 (C13), 18.7 (C1, C1′, C1″) (See Figure S2).

The mixture of 1,3-di (isoeugenyloxy)propan-2-ol and 2,3-di (isoeugenyloxy)propan-1-ol (DiEP1 and DiEP2 depicted in Scheme 1) was isolated from BioIgenol, and separated from GEiE (Scheme 1) by silica gel column chromatography on a silica gel made with cyclohexane, 10 g of raw product was added dropwise and eluted through the column using a cyclohexane/ethyl acetate mix (3:2 vol:vol) as eluent. Isolated products, and especially DiEP1 and DiEP2 where fully characterized using a cyclohexane/ethyl acetate mix (3/2 vol/vol). Isolated products, and especially DiEP1 and DiEP2 where fully characterized.

FTIR (ATR, cm^−1^): 3474 (O−H), 3019 (aromatic C−H), 1653 (C = C), 1602, 1584, 1510, 1463 (aromatic C = C).

^1^H-NMR (500 MHz, CDCl3): δ ppm 1.90 (m, H1′ and H1″), 3.42 (m, H12″), 3.85 (m, H10′, H10″), 4.13–4.26 (m, H11′, H11″, H13″), 4.41 (m, H12′), 5,74 (m, H2′, H2″ *trans*), 6.13 (m, H2′, H2″ *cis*), 6.34 (m, H3′, H3″), 6.82–6.95 (m, H5′, H6′, H9′, H5†″, H6″, H9″) (See Figure S3).

^13^C{^1^H} NMR (126 MHz, CDCl3) δ 149.8 (C8′, C8″), 147.3 (C7′, C7″), 132.3 (C4′, C4″), 130.6 (C3′, C3″), 124.3 (C2′, C2″), 118.8 (C5′, C5″), 114.8 (C6′, C6″), 109.1 (C9′, C9″), 71.5 (C12″), 71.3 (C11′), 71.0 (C12′), 69.1 (C11″), 69.9 (C13″), 55.8 (C10′, C10″), 18.4 (C1′ C1″) (See Figure S4).

Elemental analysis: C calculated 66.33%, found 64.46%. H calculated 6.78%, found 6.86%.

MS (*m*/*z*, ES+, [M + Na^+^]) calculated 407.183, found: 407.181.

##### Second Step: Synthesis of BioIgenox

**Scheme 4:**
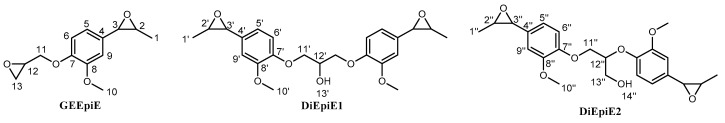
Molecular representation of BioIgenox.

BioIgenol (43.15 g, 0.16 mol, see Scheme 4), DCM (250 mL), distilled water (840 mL), sodium hydrogen carbonate (92.88 g, 1.10 mol), tetrabutylammonium hydrogen sulfate (3.76 g, 0.01 mol) and acetone (170 mL) were stirred in a 2 L round-bottom flask. Oxone^®^ (89.88 g, 0.29 mol) is gradually added during 1 h and the stirring is maintained for 4 h. Organic phase is poured in a separating funnel, washed with water, saturated NaCl solution, and dried over anhydrous MgSO_4_ leading, after evaporation under vacuum to produce a brown oil that solidifies to afford BioIgenox, as a brown waxy past (83%).

FTIR (ATR, cm^−1^): 3496 (O−H), 3027 (aromatic C−H), 1608, 1592, 1514, 1455 (aromatic C = C), 912 (epoxy ring).

^1^H-NMR (500 MHz, CDCl_3_): *δ* ppm 1.41 (m, H1, H1′ et H1″), 2.71 and 2.86 (m, H13), 2.94 (m, H2, H2′, H2″), 3.35 (m, H12), 3.46 (m, H12″), 3.51 (m, H3, H3′, H3″), 3.81 (m, H10, H10′, H10″), 3.95–4.28 (m, H11, H11′, H11″, H13″), 4.36 (m, H12′), 6.69–6.93 (m, H5, H6, H9, H5′, H6′, H9′, H5″, H6″, H9″) (See Figure S5).

^13^C {^1^H} NMR (126 MHz, CDCl_3_): *δ* 150.1 (C8, C8′, C8″), 148.1 (C7, C7′, C7″), 131.5 (C4, C4′, C4″), 118.5 (C5, C5′, C5″), 114.2 (C6, C6′, C6″), 108.7 (C9, C9′, C9″), 71.0 (C12″), 70.4 (C11′), 69.1 (C12′), 68.6 (C11), 67.0 (C11″), 59.5 (C3, C3′, C3″), 58.9 (C2, C2′, C2″), 55.9 (C10, C10′, C10″), 50.2 (C12), 44.9 (C13) 17.9 (C1, C1′, C1″) (See Figure S6).

#### 2.2.2. Curing Reaction

Polyepoxy polymers were obtained via thermal curing according to the following protocol: Diepoxy monomers containing different molar ratio of anhydride hardener were ground in a mortar at room temperature and heated (120 °C for CA and 45 °C for HHPA) until a homogeneous mixture was obtained. DMID catalyst was then added with a molar ratio of 0.025 with respect to epoxide function (ep). Polymerization reaction was studied using DSC. A few milligrams of this mixture were heated from room temperature to 180 °C at a heating rate of 5 °C min^−1^. The remaining mixture was cured in an oven at 150 °C for 2 h, before being cooled down by leaving the mold at room temperature for one hour.

## 3. Results and Discussion

### 3.1. Reactional Mechanism and Characterization of BioIgenol Mixture

**Scheme 5:**
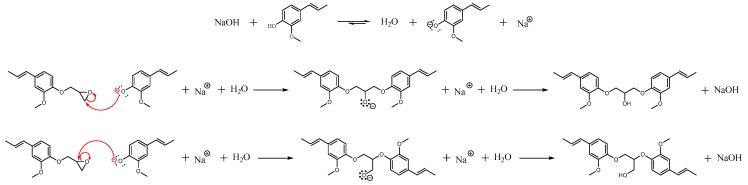
Mechanism leading to the DiEP1/DiEP2 formation.

The reaction of isoeugenol with epichlorohydrin leads, as expected, to glycidyl ether isoeugenol [21]. Two by-products, DiEP1 and DiEP2, are also formed according to the reaction mechanism shown in Scheme 5.

The phenolate anion formed in the basic conditions of the reaction acts as nucleophilic reactant toward the oxirane function of GEiE. Depending on the oxirane carbon which is attacked, DiEP1 and DiEP2 can be obtained. According to a S_N_2 mechanism occurring in the basic conditions [25] the less-substituted oxirane carbon reacts. Thus, this regioselectivity mainly leads to the formation of DiEP1. The FTIR spectrum of BioIgenol is in accordance with the proposed structures. The O–H bonds from DiEP1 and DiEP2 is observable at 3485 cm^−1^. The C=C vinyl stretching band is present at 1655 cm^−1^, and the oxirane ring from GEiE is measured at 910 cm^−1^.

DiEP1 and DiEP2 were isolated from the BioIgenol mixture by chromatography column. ^1^H NMR, ^13^C {^1^H} NMR, mass spectrometry, and elemental analysis have confirmed the formation of the dimers DiEP1 and DiEP2. In particular, ^13^C {^1^H} NMR shows five different signals attributed to the glycerol chain carbons (C11′, C12′ in DiEP1 and C11″, C12″, C13″ in DiEP2). As expected, the mass spectrum is in accordance with DiEP1 and DiEP2 structures (calculated 407.183, found: 407.181). It is noteworthy that DiEP1 and DiEP2 were not separated by a chromatography column.

### 3.2. Formation, Characterization and Curing of BioIgenox Mixture

DiEP1 and DiEP2 contain both allylic groups easily converted into epoxide functions and leading thus also to diepoxy monomers. Thus, BioIgenol mixture has been directly oxidized into BioIgenox as depicted in Scheme 2. ^1^H NMR, ^13^C {^1^H} NMR, FTIR have confirmed the formation of the three diepoxy GEEpiE, DiEpiE1 and DiEpiE2. In particular ^1^H NMR spectra show the disappearance of the allylic protons (H2, H2′, H2″ and H3, H3′, H3″ at 5.75, 6.13 and 6.34 ppm, respectively) and the appearance of the epoxy protons (H2, H2′, H2″ and H3, H3′, H3″ at 2.94 and 3.51 ppm respectively). In the ^13^C NMR spectra, the signals of allylic carbons (C3, C3′, C3″ and C2, C2′, C2″ at 130.9 and 124.6 ppm, respectively) are replaced by those of epoxy carbons (C3, C3′, C3″ and C2, C2′, C2″ at 59.5 and 58.9 ppm, respectively).

To avoid the purification of crude GEEpiE, the GEEpiE/(DiEpiE1and DiEpiE2) fractions have been estimated. Indeed the purification by chromatography of 10 g of crude GEEpiE involves an entire working day, requires more than 2 L of elution solvents and finally leads to only 6 g of pure GEEpiE.

The ^1^H NMR signals intensities allows the estimation of GEEpiE/(DiEpiE1and DiEpiE2) fractions. By measuring the intensity of the signal H12 (*I*_H12_) and total intensity of aromatic protons (H5, H5′, H5″, H6, H6′, H6″, H9, H9′ and H9″) (*I*_Ar_), the respective fractions of GEEpiE (*x*_1_) and of DiEpiE (*x*_2_ = 1 − *x*_1_) are determined by resolving the following equation:

$$\frac{I_{H12}}{I_{ar}}=\frac{x_{1}}{\left(3x_{1}+6x_{2}\right)}$$

GEEpiE was always found to be the major product of the mixture with *x*_1_ varying from 0.85 to 0.55 and on the basis of five experiments.

**Figure 1:**
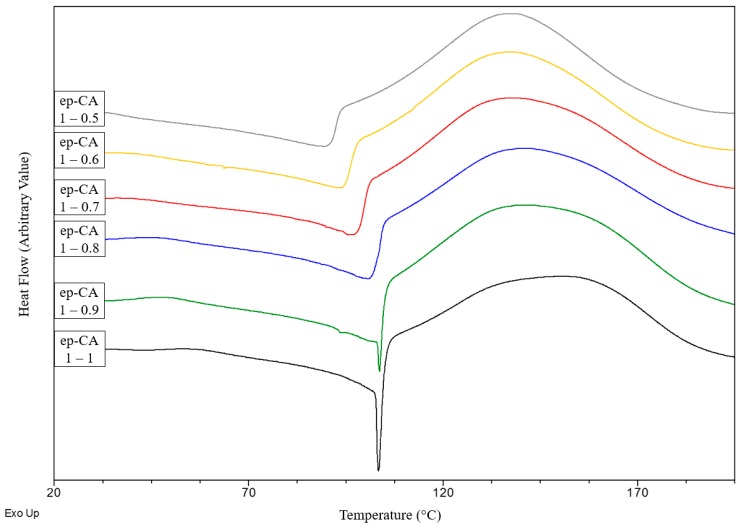
Thermograms of dynamic curing reaction of thermoset series with varying Epoxide function/CA ratios.

BioIgenox has been cured with different ratio of bio-based camphoric or hexahydrophtalic anhydride (Table 1) in order to optimize the behaviours of the resulting material. The stoichiometric epoxide function/anhydride molar ratios is 1/1. However, epoxide function/anhydride molar ratio varying from 1/0.5 to 1/1 have been investigated in order to take into account the side reactions, in particular the epoxide homopolymerization [20]. The reticulation reaction has been investigated by DSC. Samples were heated from 20 °C to 200 °C at a heating rate of 5 °C min^−1^. DSC thermograms are depicted in Figure 1.

When curing with CA, the DSC curves exhibit the maximum of the heat release associated to the reticulation reaction at 140 °C whatever the ratio. It is noteworthy that the endothermic peak at about 100 °C which is attributed to the mixture melting, decreases with the epoxide function/CA molar ratio. This is in accordance with the physical properties of the CA which is crystallized contrary to the waxy paste of BioIgenox.

**Figure 2:**
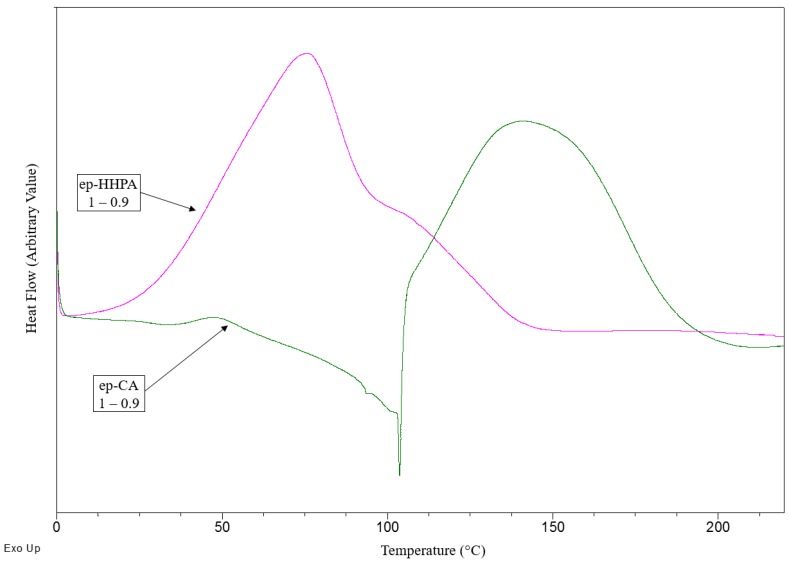
Curing of BioIgenox using CA or HHPA as a hardener.

HHPA reacts with BioIgenox at a lower temperature than CA (see Figure 2), as it was previously reported by our team in the case of GEEpiE [21,22]. Not surprisingly, no endothermic fusion peak is observed at 100 °C on the DSC thermogram. It is noticeable that these moderate curing temperatures are consistent with usual composite processing including natural fibres.

### 3.3. Properties and Characterization of BioIgenox Based Thermosets

#### 3.3.1. Effect of the Epoxide Function/Anhydride Ratio on the Thermoset’s Properties

**Figure 3:**
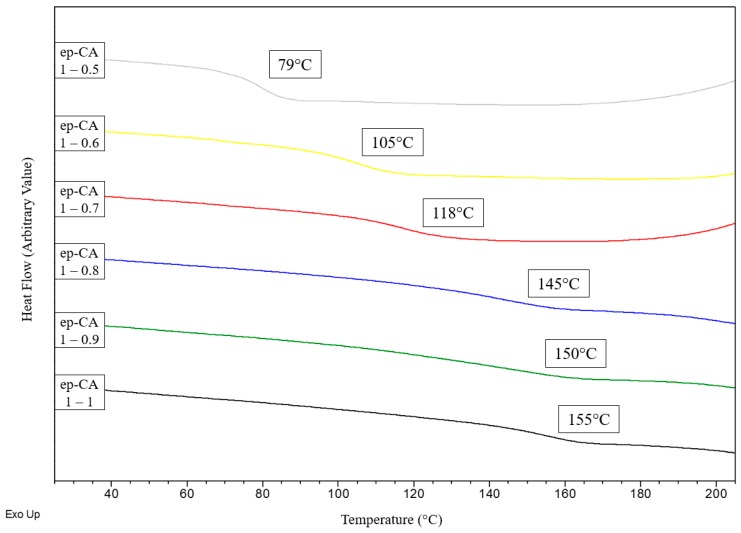
Effect of the epoxide function/CA ratio on the glass transition determined by DSC.

**Figure 4:**
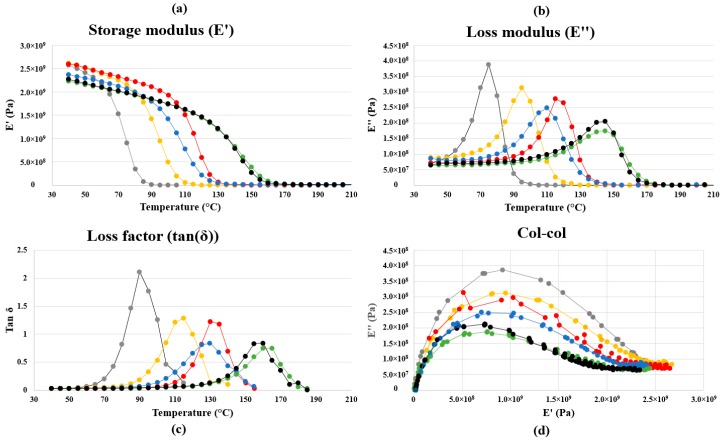
Storage modulus (**a**), loss modulus (**b**), loss factor (**c**) and Cole–Cole diagram (**d**) for ep/CA/DMIA (1/X/0.025) formulation with X = 1 (black), 0.9 (green), 0.8 (blue), 0.7 (red), 0.6 (yellow), 0.5 (grey).

The DSC curves for the BI/CA thermoset series obtained after 2 h of curing at 150 °C in oven, are shown in Figure 3 and their glass transition temperatures are reported in Table 2. As depicted in Figure 3, T_g_ significantly increases when epoxide function/CA varies from 1/0.5 to 1/0.8 while it remains quite constant for the 1/0.9 and 1/1 ratios. For the most crosslinked systems, Tg cannot be accurately determined using DSC because the change of the Cp value associated to Tg is not well pronounced. It is well known that DMA is more sensitive to the changes occurring at the Tg when compared to DSC [26,27]. Thus, DMA results were also exploited to determine the T_g_ values (Figure 4). Three different methods were used for the T_g_ determination from the DMA results, i.e., the maximum value of the *E*′ curve derivative, the peak of the *E*″ curve and the peak of the tan(δ) curve. These values, synthetized in Table 2 were determined from the raw data without any interpolation.

As usually observed, the Tg values obtained from these methods differ up to 30 °C from one another on the same run. Whatever the method used, the same tendency as for the DSC results is observed with a significant increase from 75 °C to 145 °C (when considering the peak of the *E*″ curve) when epoxide function/CA varies from 1/0.5 to 1/0.9. The change for the higher ratio is then very slight.

The evolution of the T_g_ value as a function of the level of CA certainly results from the differences in crosslinking density. Indeed, if the epoxide function/CA ratio deviates from the ideal number of reactants, it would decrease the crosslinking density and thus degrades mechanical and thermal properties. Calculated crosslinking density values from DMA data are detailed in Table 3. These values are in accordance with the augmentation of the glass transition temperature and with the decrease of the thermosets swelling ability. This is also consistent with the decrease in the maximum damping capacity observed at the Tg (see peak of the tan(δ) curve) for the increasing epoxide function/CA ratios.

Counterintuitively, the modulus slowly decreases with the augmentation of the crosslinking density. Due to this higher crosslinking density, molecules are no more able to fill free volumes, increasing the distance between neighbouring chains and reduces intermolecular interactions. This effect has been observed in non-stoichiometric epoxy/amine system, where unreacted species fill the free volume, enhancing chain interactions and increasing stiffness [28,29,30]. Furthermore, the stiffness reduction with the increasing crosslinking density is moderate in the glassy state (12% at the maximum) and the values superior to 2.2 GPa are still suitable with the development of high-grade composite materials.

Not surprisingly, swelling tests show that thermosets with epoxide function/anhydride ratios equal to 1/0.5 and 1/0.6 fragmented in small pieces in THF because of their lack of crosslinking. Other ratios swell less and less, improving the amount of hardener, i.e., the crosslinking-density, which confirm DMA results.

One can notice the decrease of the loss modulus with the increase of the ep/CA ratio (Table 3). This effect is due to the increasing crosslink density, involving a loss of ability to absorb shock. Indeed, we developed here a panel of fully bio-based materials with quasi the same modulus, but different in their ability to soften, with a glass transition temperature between 80 and 155 °C.

According the Cole–Cole diagrams, which have been traced in Figure 4, all systems present a single relaxation time.

Even if all formulations are covering the same range of storage modulus, they may be used in different applications due to their varying loss modulus (see Figure 4).

**Figure 5:**
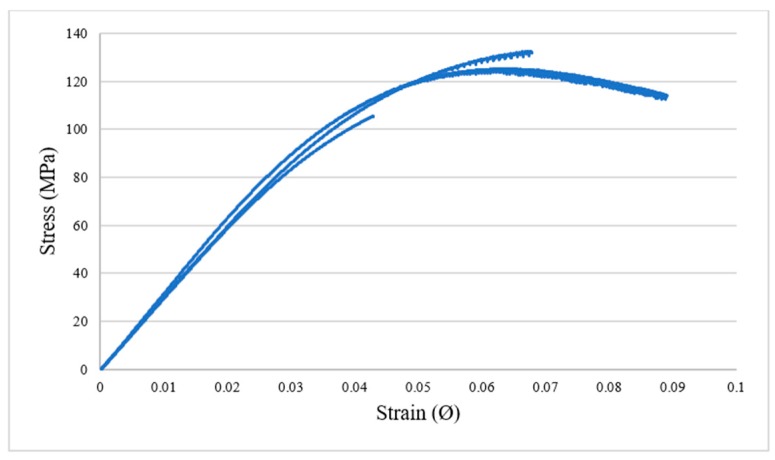
3-point bending curves for three tested specimens. Maximum bending stress vs.; strain (ep/CA/DMID 1/0.8/0.025).

For use in structural or semi-structural application, the formulation with the highest Tg are the most relevant, since it will result in the maintain of the stiffness and damping capacity when the temperature is increased. For such applications, a high bending stiffness and tenacity is also expected. three-point bending tests were performed for an epoxy material with a 1/0.8/0.025 ratio. Results are presented in Figure 5 and Table 4. This material exhibits a mostly elasto-brittle behaviour with a linear elastic response up to 3 to 4% of strain and relatively limited plastic strain range. High bending stiffness and tenacity are measured, with a mean bending stiffness of approximately 3.2 GPa and a stress and strain at failure of approximately 120 MPa and 6.6%. These values are comparable, even higher, to the one obtained with the very classical and widespread DGEBA-based epoxy systems. The measured stiffness is higher than the one measured with the DMA tests. This difference can be attributed to the inaccuracy of the stiffness measurement using DMA apparatus [31]. Indeed, it is well-known that the material stiffness is generally under-estimated in DMA, in particular due to the clamping conditions, to the strain measurement (based on the machine displacement) and to the machine compliance.

Other properties, generally expected for structural composite applications, such as impact or wear resistance, durability, fibre/matrix adhesion, gel time and pot life could be characterized in further studies to complete the technical datasheet.

#### 3.3.2. Effect of the Curing Agent on the Properties of the Thermoset

**Figure 6:**
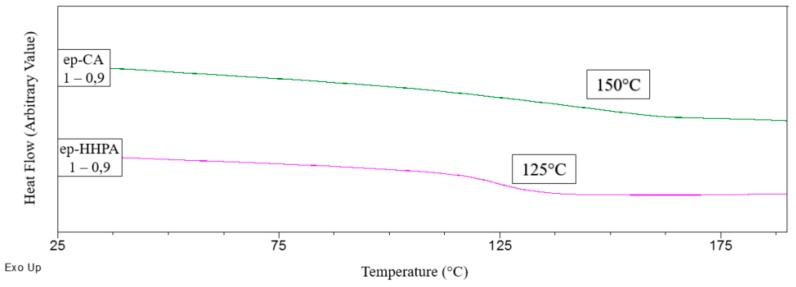
Thermograms comparison for HHPA or CA cured epoxy thermoset (ep/CA/DMID 1/0.9/0.025).

In order to extend the panel of materials available from BioIgenox, this bio-based resin has also been cured with HHPA, using the best epoxide function/anhydride molar ratio previously determined with CA hardener. The mechanical and thermal characteristics of this resulting thermoset have been compared with those obtained with the formulation involving CA hardener. DSC thermograms of both cured thermosets are depicted in Figure 6 and the mechanical properties as well as the crosslinking density values of these formulations are given in Table 5.

Even if the CA based formulation leads to a higher glass transition temperature than the thermoset cured with HHPA, the maximum damping capacity (value of tan(δ) at the peak) is lower. This may be explained by a higher disparity in chains length. Moreover, HHPA-cured thermoset has a higher modulus at 40 °C and a higher damping ability. This effect could be explained by the higher crosslinking density with a formulation involving HHPA.

These results show that both BioIgenox/CA and BioIgenox/HHPA bio-based formulations studied are promising candidates to replace DGEBA based matrices used for high-performance composites.

## 4. Conclusions

In the course of our research in the development of high-performance epoxy materials, the synthesis of a bio-based epoxy resin has been improved with the aim of making its scale-up possible. The removal of chromatography purification step has led to the obtaining of a monomers mixture named BioIgenox (BI), which has been totally characterized by spectrometric and elemental analyses.

BioIgenox and camphoric anhydride have been cured with varying epoxide function/anhydride molar ratios (1/0.5 to 1/1). Based on DMA and DSC analyses and swelling behaviours, the optimum ratio has been found to be near 1/0.9. This optimized formulation has been transposed to BioIgenox/hexahydrophtalic anhydride. Both thermosets exhibit thermal and mechanical behaviours consistent with their use as matrices for structural or semi-structural composites. Namely, BioIgenox/CA and BioIgenox/HHPA thermosets have Tg determined by DMA of 165 °C and 140 °C, respectively, and storage modulus at 40 °C of 2.2 and 2.6 GPa, respectively. The quasi-static bending properties are comparable, even higher, to the one obtained with the very classical and widespread DGEBA-based epoxy systems.

Finally, this study demonstrates an effective and ecofriendly way to prepare bio-based epoxy matrices able to replace petro-sourced DGEBA with comparable performances.

## Figures and Tables

**Table 1:** Formulation of the studied samples.

	Epoxide Function/Anhydride Molar Ratio							
	1/0.5	1/0.6	1/0.7	1/0.8		1/0.9		1/1
BI * (mg)	2.64	2.44	2.63	3.03	2.94	2.84	2.79	2.73
CA (mg)	1.57	1.72	2.16	2.86		3.01		3.26
HHPA (mg)					3.37		2.54	

* The BI sample used has a GEEpiE/DiEpiE molar ratio of 1.5/1.

**Table 2:** Glass transition temperature of epoxide thermosets.

	*E*′ Max Derivative	Max *E*″	Max Tan(δ)	Tg DSC
ep/CA	(°C)	°C	°C	Ramp 10 °C/min (°C)
1/0.5	70	75	90	80
1/0.6	85	95	115	105
1/0.7	110	115	130	120
1/0.8	110	110	130	145
1/0.9	140	145	165	150
1/1	145	145	160	155

**Table 3:** Mechanical properties of epoxy thermosets depending on the ep/CA ratio.

	*E*′ at 40 °C	Max Tan(δ)		Crosslinking Density	Swelling
ep/CA	GPa	°C *	Amplitude	mol cm^−3^	
1/0.5	2.57	90	2.11	0.70	180
1/0.6	2.62	115	1.29	2.04	79
1/0.7	2.60	130	1.23	2.43	54
1/0.8	2.41	130	0.84	3.35	55
1/0.9	2.23	165	0.75	3.66	36
1/1	2.27	160	0.84	4.58	35

* Tg is defined here as tan(δ) maximum.

**Table 4:** Three-point bending properties of epoxy thermoset (ep/CA/DMID 1/0.8/0.025).

Mean Value ± Standard Deviation	Modulus	Ultimate Stress	Strain at Failure
ep/CA	GPa	MPa	Ø
1/0.8	3.2 ± 0.1	121 ± 14	0.066 ± 0.023

**Table 5:** Properties comparison for HHPA and CA cured epoxy thermoset (ep/CA/DMID 1/0.9/0.025).

	*E*′ at 40 °C	Max Tan(δ)		Crosslinking Density
Hardener	GPa	°C	Amplitude	mol cm^−3^
CA	2.23	165	0.75	3.66
HHPA	2.59	140	0.91	4.36

## Supplementary Materials

The following are available online at https://www.mdpi.com/2073-4360/12/1/229/s1, Molecular representation with atoms labeling of BioIgenol, DiEP1, DiEP2 and BioIgenox, as well as their ^1^H, ^13^C{^1^H} NMR spectra are given in Supplementary Figures S1–S6.
