# Supplementary material for: New Eco-Friendly Synthesized Thermosets from Isoeugenol-Based Epoxy Resins
Source: Polymers (Basel). 2020 Jan 17;12(1):229. doi: 10.3390/polym12010229 (PMC7023557; doi:10.3390/polym12010229)
Supplement: Supplementary file 1 [file polymers-12-00229-s001.pdf]

New Eco-Friendly Synthesized Thermosets from Isoeugenol-Based Epoxy Resins

Quentin Ruiz [1,2], Sylvie Pourchet [1,*], Vincent Placet [2], Laurent Plasseraud [1] and Gilles Boni [1,*]

[1] ICMUB Institute, Université de Bourgogne Franche-Comté, UMR 6302 CNRS-UB, F-21000 Dijon, France; quentin_ruiz@etu.u-bourgogne.fr (Q.R.); laurent.plasseraud@u-bourgogne.fr (L.P.)
[2] FEMTO-ST Institute, Université de Bourgogne Franche-Comté, UMR 6174 CNRS-UFC-ENSMM-UTBM, Department of Applied Mechanics, F-25000 Besancon, France; vincent.placet@univ-fcomte.fr
* Correspondence: sylvie.pourchet@u-bourgogne.fr (S.P.); gilles.boni@u-bourgogne.fr (G.B.)

Figure S1. ^{1}H NMR spectrum of BioIgenol ($CDCl_3$)

Figure S2. $^{13}C\{^{1}H\}$ NMR spectrum of BioIgenol ($CDCl_3$)

Figure S3. ^{1}H NMR Spectrum of DiEP1 and DiEP2 ($CDCl_3$)

Figure S4. $^{13}C\{^{1}H\}$ NMR Spectrum of DiEP1 and DiEP2 ($CDCl_3$)

Figure S5. ^{1}H NMR spectrum of BioIgenox ($CDCl_3$)

Figure S6. $^{13}C\{^{1}H\}$ NMR spectrum of BioIgenox ($CDCl_3$)

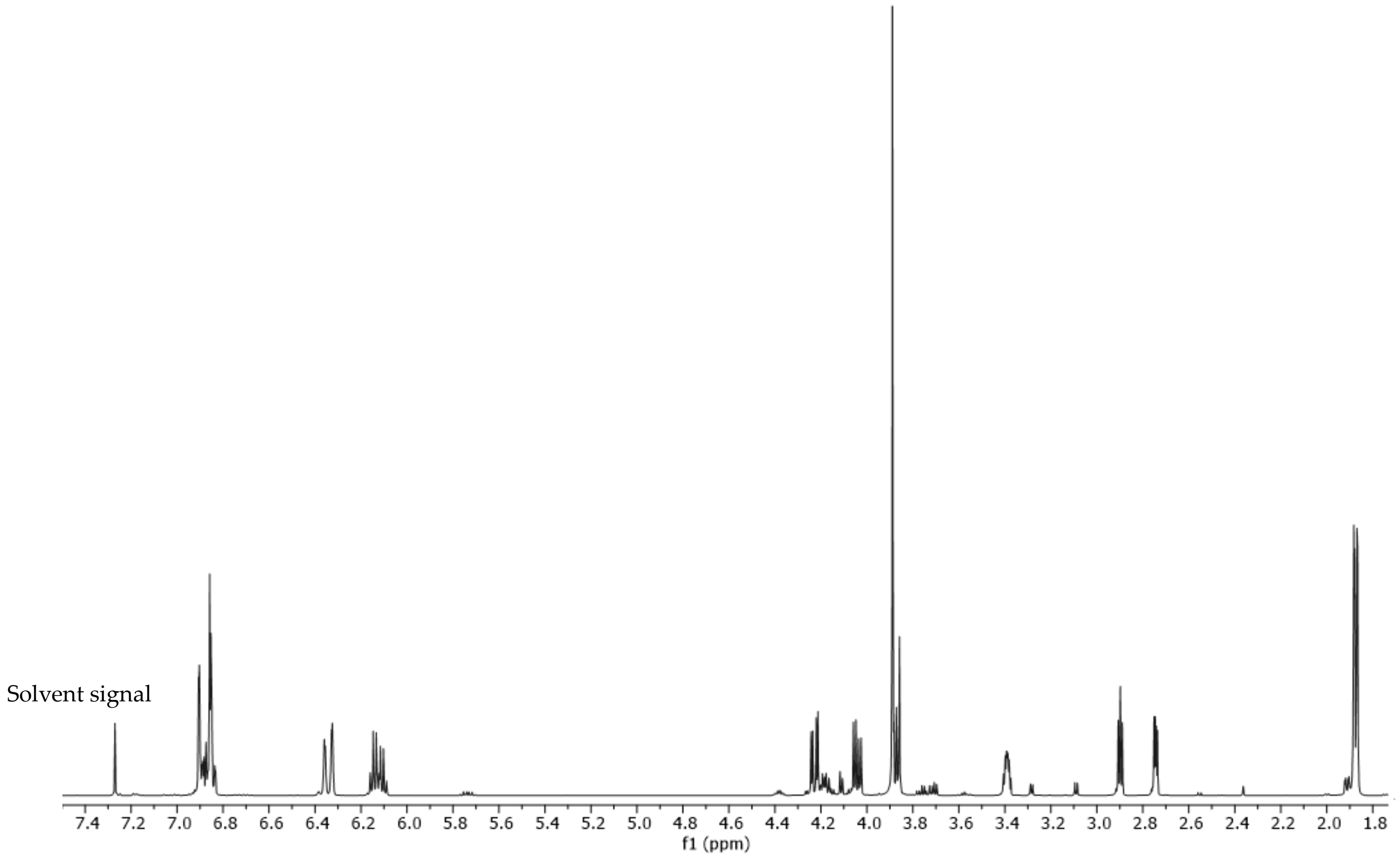

Figure S1. ^{1}H NMR spectrum of BioIgenol ($CDCl_3$)

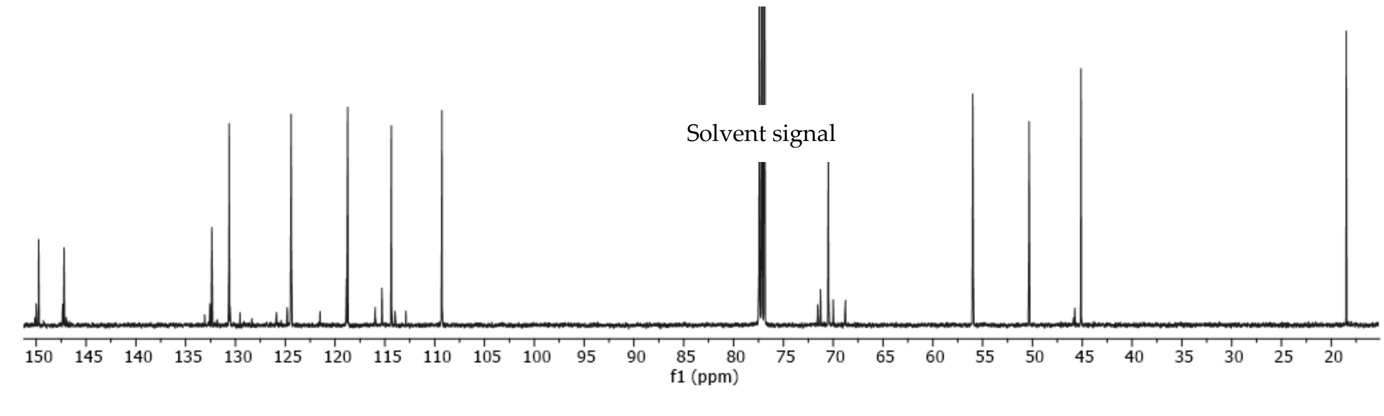

Figure S2. $^{13}C\{^{1}H\}$ NMR spectrum of BioIgenol ($CDCl_3$)

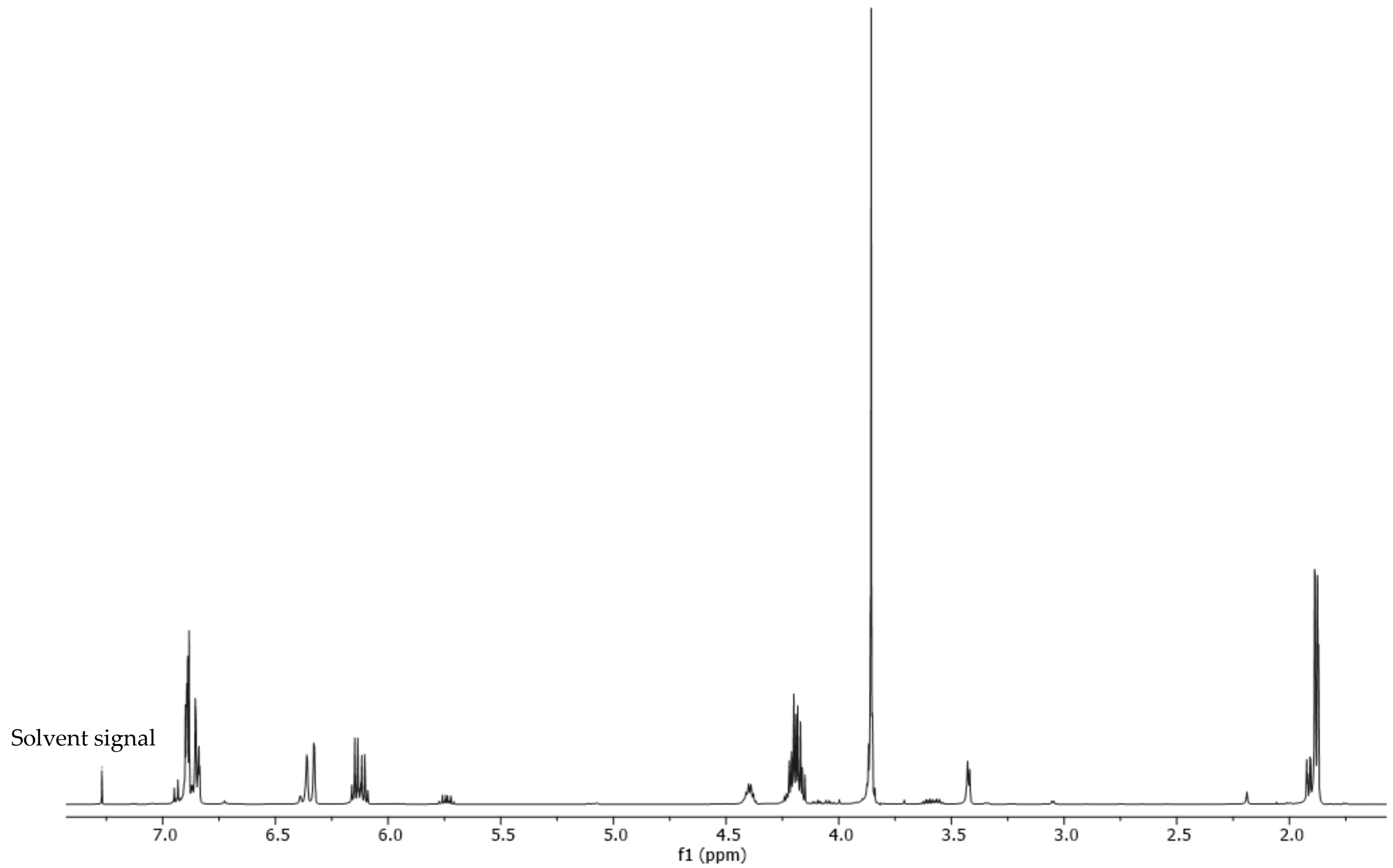

Figure S3. ^{1}H NMR Spectrum of DiEP1 and DiEP2 ($CDCl_3$)

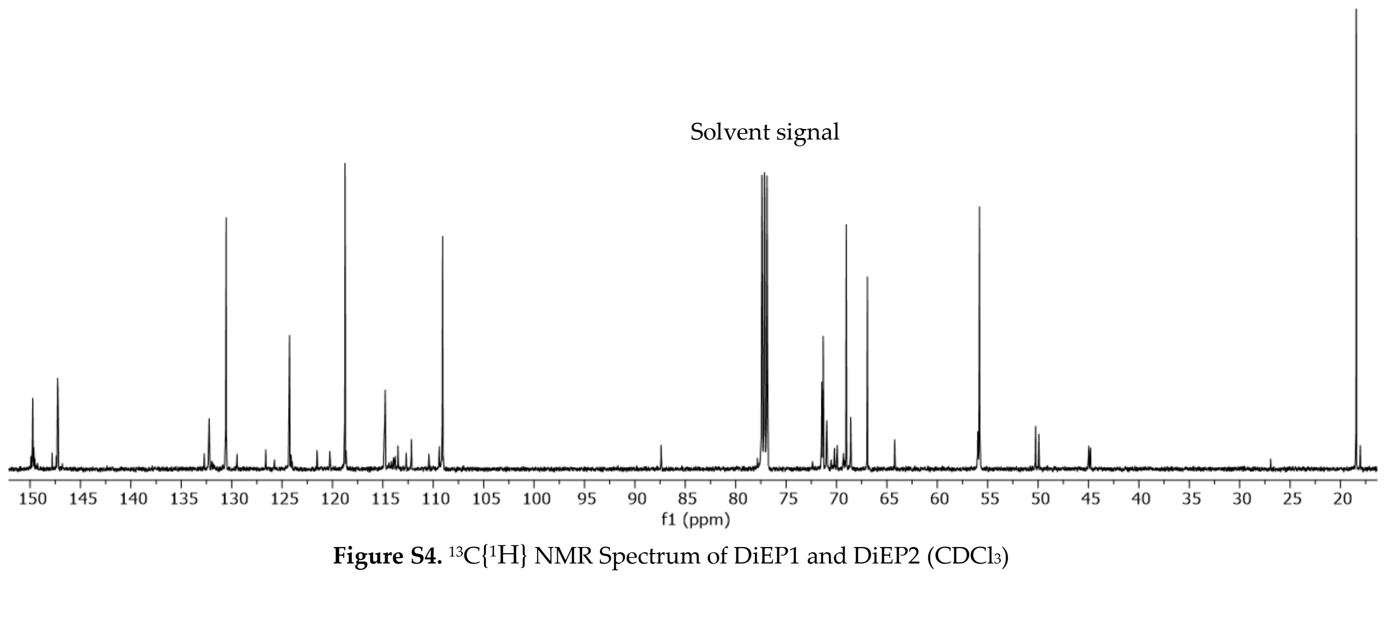

Figure S4. $^{13}C\{^{1}H\}$ NMR Spectrum of DiEP1 and DiEP2 ($CDCl_3$)

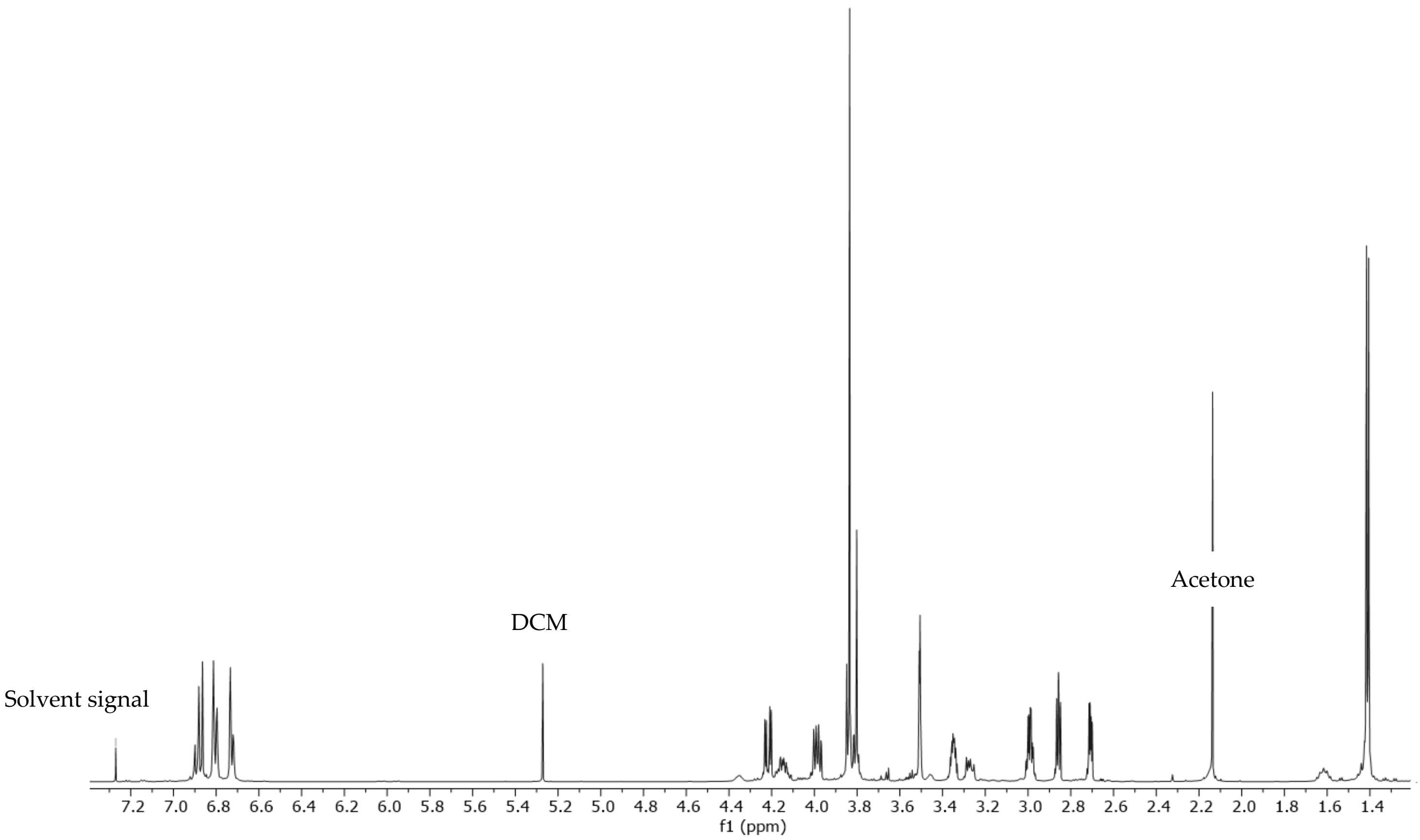

Figure S5. ^{1}H Spectrum of BioIgenox ($CDCl_3$)

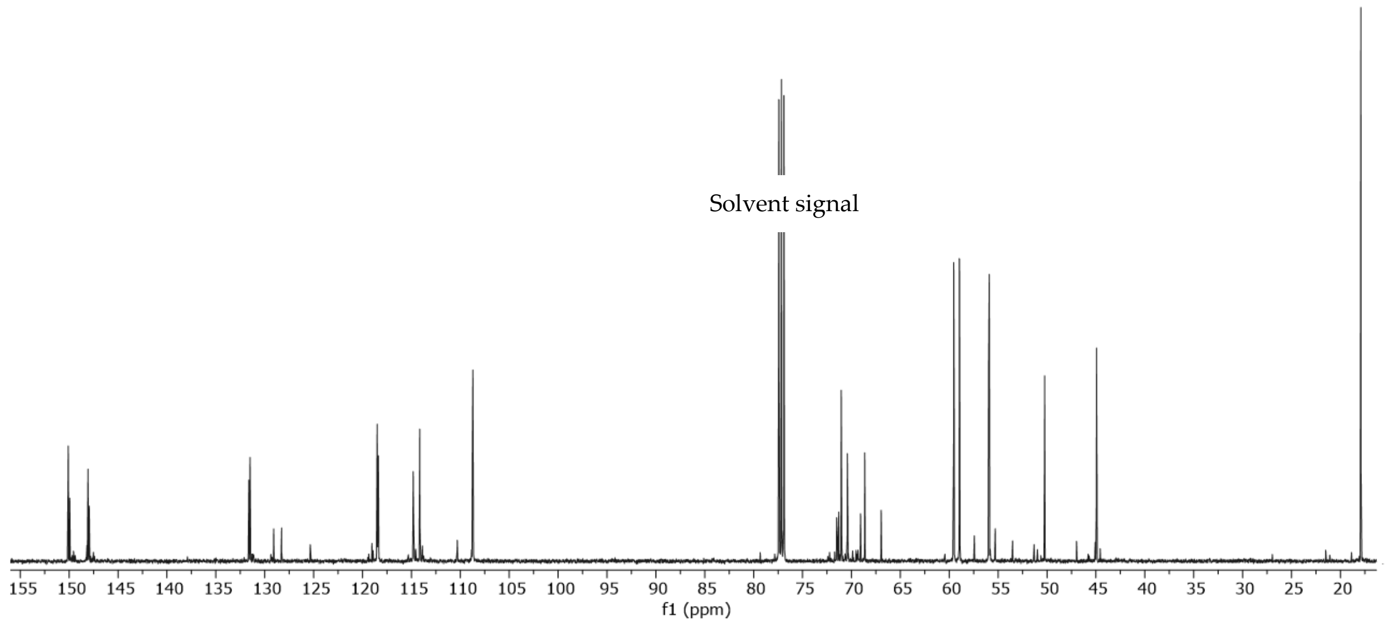

Figure S6. $^{13}C\{^{1}H\}$ spectrum of BioIgenox ($CDCl_3$)
